# Assessing and selecting gene expression signals based upon the quality of the measured dynamics

**DOI:** 10.1186/1471-2105-10-55

**Published:** 2009-02-10

**Authors:** Eric Yang, Ioannis P Androulakis

**Affiliations:** 1 Biomedical Engineering Department, Rutgers University, Piscataway, New Jersey, USA

## Abstract

**Background:**

One of the challenges with modeling the temporal progression of biological signals is dealing with the effect of noise and the limited number of replicates at each time point. Given the rising interest in utilizing predictive mathematical models to describe the biological response of an organism or analysis such as clustering and gene ontology enrichment, it is important to determine whether the dynamic progression of the data has been accurately captured despite the limited number of replicates, such that one can have confidence that the results of the analysis are capturing important salient dynamic features.

**Results:**

By pre-selecting genes based upon quality before the identification of differential expression via algorithm such as EDGE, it was found that the percentage of statistically enriched ontologies (p < .05) was improved. Furthermore, it was found that a majority of the genes found via the proposed technique were also selected via an EDGE selection though the reverse was not necessarily true. It was also found that improvements offered by the proposed algorithm are anti-correlated with improvements in the various microarray platforms and the number of replicates. This is illustrated by the fact that newer arrays and experiments with more replicates show less improvement when the filtering for quality is first run before the selection of differentially expressed genes. This suggests that the increase in the number of replicates as well as improvements in array technologies are increase the confidence one has in the dynamics obtained from the experiment.

**Conclusion:**

We have developed an algorithm that quantifies the quality of temporal biological signal rather than whether the signal illustrates a significant change over the experimental time course. Because the use of these temporal signals, whether it is in mathematical modeling or clustering, focuses upon the entire time series, it is necessary to develop a method to quantify and select for signals which conform to this ideal. By doing this, we have demonstrated a marked and consistent improvement in the results of a clustering exercise over multiple experiments, microarray platforms, and experimental designs.

## Background

As biology has transformed from a descriptive to a quantitative field, there has been growing interesting in creating mathematical models which describe the dynamic evolution of biological processes. Thus rather than taking measurements pre vs. post perturbations, there has been growing interest in modeling the dynamic progression of biological responses [1-3]. However, given the exigencies of biological experiments, there are often limitations in the signal to noise ratio of a given measurement leading to large variations between replicate measurements as well as relatively few replicates despite the lack of precision in these measurements. Because of the desire to mathematically model the dynamics of the system, it is not appropriate to only determine whether the signal shows a statistically significant change over the given time course, but also that the dynamic variations amongst all the different time points are important.

An example of a dynamic biological signal which is of interest researchers are the changes in mRNA gene expression level over time in response to external perturbations such as gene silencing, induction of disease states, or the administration of a drug/toxin[4,5]. The study of this specific signal has evolved from determining which systems show statistically significant changes, to modeling the progression of this change to obtain intuitions about the underlying mechanisms. An example of this would be the use of temporal gene expression profiles to probe the underlying mechanism which governs the PK/PD response of an organism to a drug, or the dynamic response of an organism in response to a severe injury[6].

**Figure 1:**
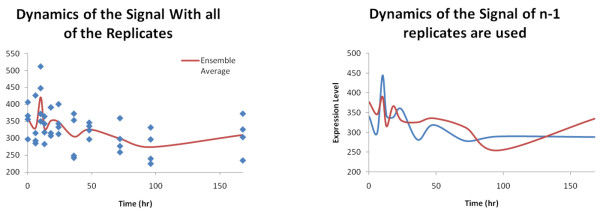
**Given the limited number of replicates for a given experiment, it is difficult to determine whether sufficient replicates have been captured**. Had three replicates rather than four replicates been used, the captured signal could have been significantly different. In this case, the sample with three replicates randomly drops one of the replicates in the.

The standard procedure for obtaining the necessary information consists of taking a set of gene expression measurements at predetermined time points and reconstructing the dynamic signal. Due to the low signal to noise ratio associated with these experiments, replicates are taken in order to compensate for the lack of fidelity in the signals. However, because of issues such as cost in terms of time and money, oftentimes very few replicates are obtained at each time point. Therefore, while it may be relatively simple to determine whether the system changes in a measurable fashion during the time horizon of the experiment via statistical tests such as ANOVA[7], t-test[8], EDGE[9] or SAM[9], determining whether the dynamic of the response of the system has been accurately captured is a problem which has been less well addressed. We propose the creation of an algorithm formulated specifically to address this issue. The significance of this difference can be illustrated in Figure 1. Figure corresponds to the gene expression profile of PRP8 pre-mRNA processing factor 8 in rat which is present in the Gene Expression Omnibus dataset under accession number GDS972[10]. This gene contains four replicates per time point. In the expression profile for this specific gene, it is possible to determine via an EDGE whether the gene has been differentially expressed over the experimental time course. However, depending upon which subset of 3 replicates is selected, the dynamic response of the gene expression profile appears to change significantly. Therefore, the question which we need to answer is whether sufficient replicates have been measured such that the dynamics which will later be fitted via various mathematical models represents the underlying response.

Therefore, the question is not how to select for biologically relevant signals, but rather how to select for the signals whose inherent expression dynamics given the large biological variance and the limited number of replicates is accurately captured by the ensemble average. While there exist several methods for assessing the quality of a signal given a limited number of replicates, such as calculating the Signal-to-Noise Ratio (SNR)[11] or utilizing the co-efficient of variance (CV)[1], these methods have significant drawbacks. The two primary drawbacks with these methods is that they are insensitive to the number of replicates used within a given experiment, and secondly while one can easily set a cut-off for a given score, it is difficult to interpret the meaning of this cutoff statistically i.e. associating the cut-off with a p-value.

As a generalized method for assessing the quality of a single temporal signal given a specific number of replicates, we need to satisfy two intuitions:

1. The accuracy of the ensemble average increases as the number of replicates per time point increases

2. The accuracy of the ensemble average increases as the coefficient of variance decreases at each time point

## Results

**Figure 2:**
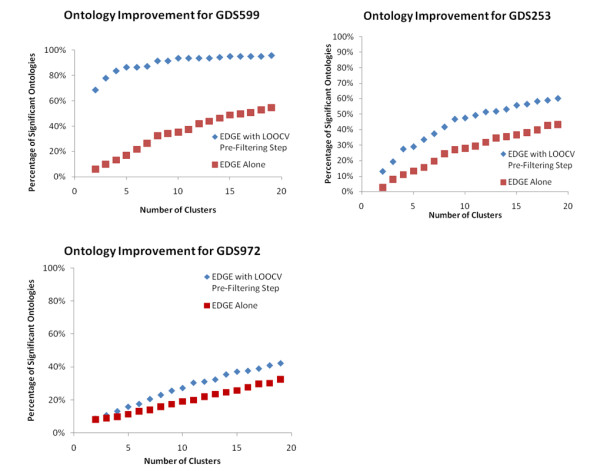
**The improvements observed over three different datasets when using the LOOCV Quality Assessment metric vs. EDGE**. In all cases, there exists a consistent improvement with the LOOCV method, but advances in both technology or increasing the number of replicates will close the gap between the two methods as evidenced by GDS972, a dataset measuring chronic infusion of datasets run on the RAE230A array with 4 replicates per time point.

For all of the datasets, the p-value cutoff was selected at p < .05 for both the EDGE as well as the LOOCV Quality Assessment. While it is arguable as to whether such a threshold is appropriate given the number of genes present within the dataset[12], what we seek to show is that for a given threshold that filtering genes based upon the accuracy as well as differential expression exhibits a stronger link between co-expression and co-regulation than merely selecting the genes based upon their differential expression via algorithms such as EDGE. For all of the datasets, the selection of genes based upon the quality of their dynamic expression profiles showed this consistent trend as exemplified by the greater percentage of statistically enriched ontologies Figure 2. This matches well with our original hypothesis that if we were to cluster genes whose measured dynamics were more accurately captured, then the association between co-functionality and co-expression becomes stronger. Thus through the LOOCV pre-filtering step, we see a decrease in the number of genes which have been included, but which do not truly correlate with the genes in a given cluster.

Looking at the results for the three datasets in Table 1, we can see that the increase in the percentage of significantly enriched ontologies is due primarily to the fact that the number of non-significantly enriched ontologies is decreasing, while the number of significantly enriched ontologies remains relatively constant. Therefore, the function of the LOOCV algorithm seems to be the removal of genes which do not appear to show significant co-functionality with the other genes they are grouped with. Looking at the intersection of

**Table 1:** Tabulates the total number of ontologies that were found for a given number of clusters for each dataset, as well as the number of significantly enriched ontologies.

		Edge Only		LOOCV + EDGE	
Gene Set	Clusters	Significant Ontologies	All Ontologies	Significant Ontologies	All Ontologies

GDS253	2	42	1696	136	1049
	
	3	133	1696	202	1049
	
	4	183	1696	288	1049
	
	5	222	1696	304	1049
	
	6	261	1696	352	1049
	
	7	330	1696	392	1049
	
	8	441	1696	438	1049
	
	9	455	1696	491	1049
	
	10	470	1696	499	1049
	
	11	497	1696	517	1049
	
	12	537	1696	540	1049
	
	13	584	1696	544	1049
	
	14	598	1696	558	1049
	
	15	620	1696	585	1049
	
	16	642	1696	593	1049
	
	17	673	1696	612	1049
	
	18	721	1696	619	1049
	
	19	733	1696	632	1049

GDS599	2	78	1292	96	140
	
	3	129	1292	109	140
	
	4	172	1292	117	140
	
	5	219	1292	121	140
	
	6	282	1292	121	140
	
	7	344	1292	122	140
	
	8	420	1292	128	140
	
	9	444	1292	128	140
	
	10	456	1292	131	140
	
	11	484	1292	131	140
	
	12	544	1292	131	140
	
	13	569	1292	131	140
	
	14	600	1292	132	140
	
	15	632	1292	133	140
	
	16	644	1292	133	140
	
	17	658	1292	133	140
	
	18	684	1292	133	140
	
	19	708	1292	134	140

GDS972	2	189	2372	98	1696
	
	3	211	2372	133	1696
	
	4	230	2372	183	1696
	
	5	265	2372	222	1696
	
	6	310	2372	261	1696
	
	7	330	2372	330	1696
	
	8	374	2372	411	1696
	
	9	413	2372	455	1696
	
	10	450	2372	470	1696
	
	11	471	2372	497	1696
	
	12	521	2372	537	1696
	
	13	558	2372	584	1696
	
	14	582	2372	598	1696
	
	15	609	2372	620	1696
	
	16	656	2372	642	1696
	
	17	707	2372	673	1696
	
	18	714	2372	721	1696
	
	19	773	2372	733	1696

In all of the cases, we see that after running the LOOCV filter as well as the selection via EDGE, the total number of ontologies that have been selected is lower, whereas the number of statistically significantly enriched ontologies remains relatively constant. Thus, the improvement in the total number of ontologies appears to be due primarily to the removal of genes that do not show significant co-functionality.

For the GDS972 chronic corticosteroid dataset, we see the smallest amount of improvement between filtering the dataset utilizing an EDGE vs. utilizing EDGE along with the proposed LOOCV filtering algorithm. The pre-selection step via LOOCV yielded 2776 genes, of which EDGE determined that 2127 of them were differentially expressed. This is in contrast to running EDGE independently in which 5344 genes were selected as being differentially expressed. In this instance, it would appear that filtering via LOOCV identifies a subset of genes in which over 75% of the genes show significant differential expression. In terms of the gene ontology enrichment, it is evident in Figure 2, that there is a consistent improvement in the percentage of significantly enriched ontologies when EDGE is used in conjunction with LOOCV.

The burn dataset (GDS599), showed the greatest improvement when the LOOCV algorithm was used as pre-filtering step. As a result of the selection via EDGE 1292 genes were selected as being differentially expressed. The result of running the LOOCV algorithm by itself yielded 644 genes, out of which 396 were selected to be differentially expressed under EDGE. Pre-filtering this dataset for quality before conducting selection for differential expression showed the greatest level of improvement due to the fact that it contained the fewest number of replicates as the fact that it was run on an older array Figure 2.

For the acute corticosteroid dataset (GDS253), after the initial filtering via LOOCV, there existed 898 genes, of which 820 were shown to be differentially expressed via EDGE. When conducting the selection via EDGE itself, 2267 genes were denoted as being differentially expressed In Figure 2, we can again see a notable and significant improvement in the percentage of significantly enriched ontologies present within the data. The GDS253 data shows an intermediate level of improvement with respect to the other datasets. This dataset was expected to show a lesser degree of improvement than the burn dataset (GDS599) due to the fact that it was run with more replicates per time point, and was expected to show a greater degree of improvement than dataset corresponding to the chronic infusion of corticosteroids (GDS972) because it is an older array (RG-U34A vs. RAE230A), and because it has fewer replicates per time point than the GDS972 dataset.

In general, for all of the datasets, the majority of genes which were selected as to having their dynamics being reliably measured also showed significant activation though the reverse is not true. This is not surprising because the LOOCV quality assessment requires that the presence of a change or lack of change to be consistent for all data points, whereas techniques such as the EDGE only attempt to detect a significant change over the time course of the experiment. However, with a sufficient number of replicates or an increase in the signal to noise ratio, both sets should be essentially the same as seen in the dataset associated with a chronic administration of corticosteroids. However, in the cases where the number of replicates is quite small or the system has a low inherent signal to noise ratio, the differences can be significant.

Given the fact that the number of significantly enriched ontologies appears to be reasonably constant whether we use the pre-filtering step or not, one may assume that the intersection of the significantly enriched ontologies between the two cases is quite high. However, from our results, this did not appear to be the case. Running the pre-filtering step along with EDGE would yield 55–60% commonality in terms of the significantly enriched ontologies identified for the two corticosteroid related datasets GDS972 and GDS253. In the case of the burn dataset GDS599, it was found that the commonality between the two sets changed from 60% when 2 clusters were used to less than 10% when 19 clusters were used. Furthermore, not all of the ontologies found after running LOOCV and EDGE were found to be enriched when EDGE was run by itself. Given the large percentage of genes which passed the LOOCV pre-filtering step which also showed differential expression, this result appears to suggest that the enrichment of individual ontologies can be quite sensitive to the incorporation or removal of a few genes.

## Discussion

The primary issue which this algorithm was developed to address was the fact that just because a gene shows significant changes in its temporal expression does not mean that such a gene expression profile has been measured in such a way which is amenable to mathematical modeling. Given the difficulty in quantifying the accuracy of a given mathematical model, clustering was used as a surrogate. In the dataset which was obtained with a newer Affymetrix array and a higher number of replicates, we generally found that genes which showed significant activation were also very likely to have been accurately measured. However, for some of the older arrays, we have found that this was not to be the case. In one case, we found that many of the genes which had been reported as having statistically significant changes in activity did not have profiles which were amenable to modeling.

Aside from the simple explanation that such variability between the replicates is due biological variability, we hypothesize that other factors may play a role and by identifying these factors, we can minimize the variations between samples. Such factors may include issues with the microarray itself as evidenced by the minimal difference between the proposed LOOCV quality assessment metric and the EDGE when utilizing the newer RAE230A array vs. the older RG-U34A arrays. Other factors which may play a role is the imperfect synchronization between replicates, especially for genes with quick early responses[13]. Thus, while researchers have attempted to balance the number of time samples taken and the presence of early, rapidly varying signals, additional care may be needed to carefully synchronize the systems to minimize the effect of incorrectly synchronized replicates adding significant variability into each replicate.

**Figure 3:**
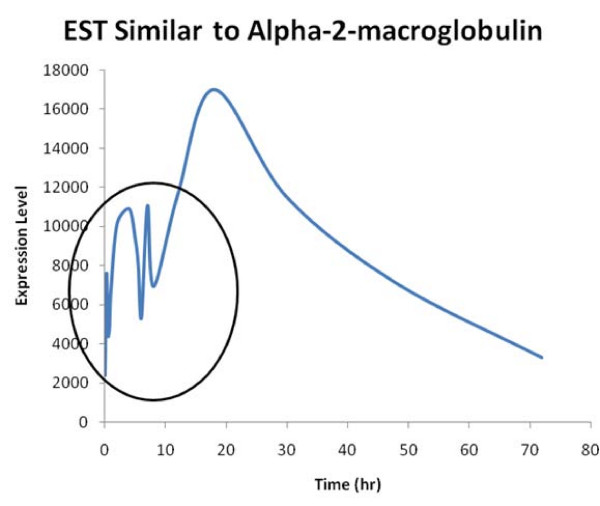
**For the GDS523 dataset associated with an acute administration of corticosteroids, the early time points are associated with rapid dynamics which due to the greater number of samples, may adversly affect the correlation coefficient, despite the fact that the majority of the experimental duration, the signal has been accurately measured**.

Due to uneven temporal sampling, one significant issue has arisen, specifically how to deal with the samples which encompass a shorter time duration vs. samples that represent the response over a longer duration of time. For instance in the case of the GDS253 dataset, the sampling rate ranges from 15 minutes to 24 hours. Thus while, the majority of the signal in terms of duration of time may have been well captured, the overall correlation coefficient may be low given the high variability in the early time points Figure 3. This is a problem which not only affects the proposed algorithm, but also many other algorithms designed to processes high throughput gene expression data.

The reason for this problem is the fact that the algorithms essentially treat the data as a vector of values without time dependence. Essentially the data points themselves are all given equal weight whether they take place during a short period of time, or whether the data point encompasses a greater period of time. Thus the correlation coefficient or clustering analysis may not also agree with one's judgment utilizing visual inspection of the data. However, while the results of the algorithm may not agree with one's intuition when visually assessing the data, the fact that researchers have selected such an uneven sampling strategy means that the dynamics early may play just as important role as the later dynamics despite their transient effect. Therefore, while there exists algorithms that will normalize the data based upon the time duration via techniques such as interpolation or curve fitting[14], they may miss or minimize the fact that earlier time points may in fact be more important biologically. Despite the ambiguities in how the uneven sampling should be treated, our framework opens up a possible extension which can address this issue. Rather than taking the ensemble average of a subset of replicates, we can interpolate/fit curves from the data with even sampling points, and then calculate the correlation coefficients from these curves and utilize the correlation coefficient in the same manner as presented.

While the intent of our algorithm was to identify a set of gene expression profiles whose temporal profiles are amenable to modeling, we assessed the effect of these high quality expression profiles through an analysis of clustering results. In our evaluation of clustering quality, we have established the fact that our algorithm identifies a condensed set of genes which show a strong co-functionality/co-expression relationship, and rejects many genes which do not show co-functionality with the dominant biological processes due to incorrect cluster assignments due to ambiguities in the underlying signal. However in many cases, this specificity comes at the expense of generality, with some of the results exhibiting fewer total enriched gene ontologies. Thus, if one wanted to use this reduced set of genes as a representative population, one important question is whether this smaller set of reduced ontologies exhibit a more focused set of biological processes or whether there is significant amount of information which has been rejected.

To make this assessment, we evaluate the set of genes that have passed EDGE, but rejected by the LOOCV algorithm, and the set of genes that pass both filters. When this evaluation is performed upon our three datasets, we see an interesting result. In the set of differentially expressed genes which **do not **pass the LOOCV filter, we see that the majority of enriched ontologies are the same as the overall set of differentially expressed genes (~95% similarity). In the cases that additional ontologies are found in this set, very few of the additional biological processes have been previously associated with injury, inflammation, the immune response, or metabolism which are hallmarks of burn injury or corticosteroid administration. This is in contrast to the set of differentially expressed genes which **do pass **the LOOCV filter. In this case, many additional biological processes were found to be over-represented. Furthermore, these additional processes have been linked to our experimental perturbations Table 2(a–c). Because of the high number of additional processes which appear to be enriched under the LOOCV case, as well as their relation to the experimental conditions, we hypothesize that this smaller set is in fact more targeted.

**Table 2:** Enriched ontologies that were not present in the original EDGE selection, but appeared when examining the subset that did not pass LOOCV after EDGE, and the subset that passed both LOOCV and EDGE.

a	
GDS599	

Rejected by LOOCV	Accepted by LOOCV

actin polymerization or depolymerization	cellular carbohydrate metabolic process
amino acid biosynthetic process	cellular alcohol metabolic process
coenzyme biosynthetic process	response to external stimulus
ER to Golgi vesicle-mediated transport	response to stress
fat-soluble vitamin metabolic process	response to stimulus
nucleobase, nucleoside, nucleotide and nucleic acid transport	generation of precursor metabolites and energy
	defense response
	inflammatory response
	response to wounding
	acute inflammatory response
	acute-phase response

	
b	

GDS253	

Rejected by LOOCV	Accepted by LOOCV

blood vessel remodeling	acute-phase response
cholesterol transport	alcohol biosynthetic process
endothelial cell proliferation	coenzyme biosynthetic process
keratinocyte differentiation	cofactor biosynthetic process
positive regulation of epithelial cell proliferation	DNA damage response, signal transduction
regulation of heart contraction	gluconeogenesis
regulation of muscle contraction	hexose biosynthetic process
response to hydrogen peroxide	monosaccharide biosynthetic process
sterol transport	purine ribonucleotide biosynthetic process
	regulation of circadian rhythm
	ribonucleotide biosynthetic process
	translational initiation

	
c	

GDS972	

Rejected by LOOCV	Accepted by LOOCV

ameboidal cell migration	activation of immune response
base-excision repair	activation of plasma proteins during acute inflammatory response
DNA-dependent DNA replication	aging
ER to Golgi vesicle-mediated transport	alcohol catabolic process
fatty acid beta-oxidation	ATP synthesis coupled electron transport
fatty acid oxidation	B cell mediated immunity
germ cell migration	bile acid metabolic process
Golgi vesicle transport	carbohydrate catabolic process
I-kappaB kinase/NF-kappaB cascade	cellular aromatic compound metabolic process
modification-dependent macromolecule catabolic process	cellular carbohydrate catabolic process
modification-dependent protein catabolic process	cofactor biosynthetic process
proteasomal ubiquitin-dependent protein catabolic process	complement activation
protein amino acid N-linked glycosylation	complement activation, classical pathway
regulation of cellular biosynthetic process	DNA damage response, signal transduction
regulation of DNA replication	DNA damage response, signal transduction by p53 class mediator
regulation of protein import into nucleus	gas transport
ubiquitin-dependent protein catabolic process	glucose catabolic process
	glutamine family amino acid catabolic process
	glycolysis
	heterocycle metabolic process
	hexose catabolic process
	humoral immune response mediated by circulating immunoglobulin
	immunoglobulin mediated immune response
	lipid biosynthetic process
	lymphocyte mediated immunity
	mitochondrial ATP synthesis coupled electron transport
	monosaccharide catabolic process
	oxidative phosphorylation
	protein targeting to mitochondrion
	response to toxin
	S-adenosylhomocysteine metabolic process
	steroid biosynthetic process
	sulfur compound biosynthetic process

It is evident that in all cases the subset that passed both LOOCV and EDGE introduced more additional ontologies. Furthermore, one of the interesting observations is that through the rejection of a population of genes, we are better able to see evidence of biological processes that are associated with inflammation, the immune response, metabolism, and injury (red) which are hallmarks of our experiments associated with the anti-inflammatory effects of corticosteroids or the response to a significant burn injury.

From this result, it appears that the set of genes rejected by the LOOCV filter are qualitatively similar to the original set of differentially expressed genes. This is in contrast to the set of differentially expressed genes which pass the LOOCV filter, which show significant differences in the identified ontologies. By looking at the set of ontologies which have identified after LOOCV filtering, but not present under selection via EDGE, it appears that LOOCV filtering is able to identify ontologies which predominately relate to the biological processes associated with our experimental perturbations. However, because it is difficult to assess whether "unrelated" ontologies reflect an artifact of the selection/clustering/enrichment process, or part of important, but previously uncharacterized processes, one strategy may be to utilize a union of both the results obtained from EDGE filtering and set of enriched ontologies after the additional LOOCV filtering. Similar to the fact that LOOCV was designed as an addendum to gene selection algorithms to identify high quality temporal profiles for modeling, the additional ontologies that have been identified serve as an addendum to the original processes identified via the original gene selection process. Because we have shown that these additional processes are relevant, we see this as adding information to what has been previously identified. Thus, the LOOCV filtering step should not supplant results from EDGE or any other selection algorithm, but can be used to complement the original results.

## Conclusion

One of the primary motivations for creating a new way of performing gene selection is that given inherent biological variability as well as deficiencies in measurement precision, the temporal evolution of a given piece of data may not be an accurate reflection as to the underlying dynamics. Therefore, if one were to mathematically model a given dynamic response, one must be certain that the data is sufficiently precise. Given the difficulties in evaluating the change in utility between modeling accurately vs. inaccurately measure data, the effect upon ontology enrichment was used instead, and we found that in all cases, there was an improvement in the overall quality of the clustering results, though with better data acquisition platforms and experimental design this improvement was minimized.

Though most of the analysis has been performed upon microarray data, this data was selected primarily for the ease by which it would be possible to evaluate the improvement, this technique can be expanded to other data types, and evaluate whether sufficient data has been obtained to for modeling purposes. Thus, this technique can be expanded for use in techniques such as ELISA over multiple time points or metabolite measurements over multiple time points, and not just microarray data, thus allowing the researcher to determine that a sufficient number of replicates has been obtained, or that more replicates are needed.

## Methods

To satisfy these constraints we propose utilizing a variation of the Leave One Out Cross Validation (LOOCV)[15] technique in which at every time point, either the maximum or the minimum point is removed, and a new ensemble average is calculated. Normally, LOOCV, a specific case of k-fold cross validation is utilized to minimize the degree of over-training or over-fitting of a given classifier or an underlying mathematical model. However, rather than determining whether a given model properly explains the data, we seek to measure the inverse; whether the data reflects the dynamics of some underlying though unknown model. Thus, given the amount of noise present in biological experiments, we seek to verify that sufficient number of replicates were obtained to properly capture the underlying signal rather than noise.

Though there are classes of mathematical models such as b-splines[16] or auto-regressive (AR)[17] models which can be used to fit the data, and therefore be used as a basis for the LOOCV analysis, each of them requires some *a priori *knowledge about the dynamics themselves. For instance when utilizing b-splines, one needs to specify the number of knots or control points to be used by the spline. In the case of AR models, the order of the model must be specified *a priori*. In both of these methods, the specification of these parameters will have a significant effect upon how the data is fitted by the model, and therefore a significant effect upon the estimation of how accurate the measured data reflects the underling dynamic. Therefore, we seek a method which is independent of model parameters, and is dependent only upon the confidence interval selected by the researcher.

### Leave one out cross validation (LOOCV)

Ideally, we would like to predict whether utilizing an additional replicate for each time point would be change the gene expression profile obtained. While we cannot predict the effect of having an additional replicate, we can simulate the effect by measuring the stability of the signal given n-1 replicates. Thus, treating the ensemble average of a temporal signal as the model, we essentially are evaluating whether taking a subset of the measured data, reflects a similar underlying model. Because the algorithm evaluates a sub-sampled signal utilizing n-1 replicates, this is similar to LOOCV in which one attempts to determine whether a given model can predict the occurrence of a data point which was not utilized in the original training.

**Figure 4:**
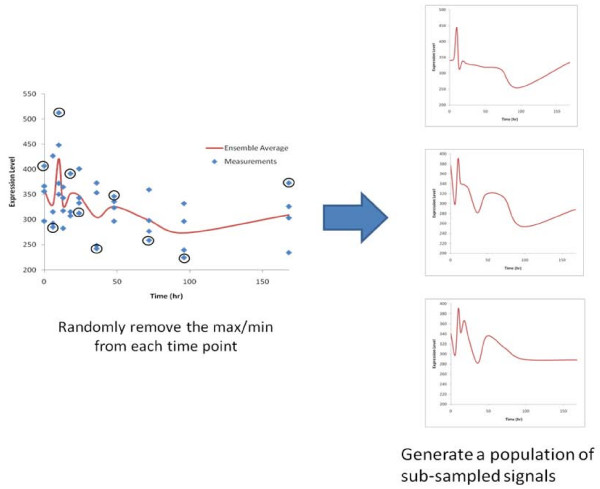
**The method for generating all of the different sub-sampled signals**. For each time point, the maximum or the minimum replicate is randomly removed. The ensemble average is then taken of these sub-sampled signals. A population of these sub-sampled signals are then generated for a similarity comparison.

Rather than performing the standard LOOCV in which a point is randomly removed from the dataset, we will remove either the minimum or maximum at each time point. Given the small number of replicates per time point, we have elected to use this strategy shown in Figure 4, to maximize the difference between the different sub-sampled signals. Because of this, a signal with length 4 will have 2^4 ^or 16 possible sub-sampled signals, a signal with length N will have 2^N ^possible sub-sampled signals. A sub-sampled signal of length four could have the maximum data value removed at time points 1,3,4 and the minimum data value removed at time point 2. This signal would be compared to its inverse which is a signal generated by the removal of the minimum data value at time points 1,3,4 and the maximum removed at time point 2. By iterating through all possible sub-sampled signals, we can establish a lower bound to the quality of a given signal.

### Similarity measure

Given the ability to generate hypothetical gene expression profiles utilizing n-1 replicates, it is then necessary to quantify the difference between these hypothetical signals. To do so, we have utilized Pearson's correlation **(1) **as a method for assessing similarity. Pearson's correlation was selected over other similarity measures because it is scale invariant allowing the comparison of signals of different magnitude. One of the inherent requirements for utilizing the Pearson's correlation for assessing the similarity between two datasets is that they need to be linearly correlated. However, because we are assessing whether different sub-sampled signals are sufficient in capturing the same underlying dynamic, it follows that the different sub-sampled signals will be linearly correlated.

Furthermore, the use of Pearson's correlation is attractive because the R^2 ^correlation coefficient associated with it can easily be converted into an s-value via Error! Reference source not found. which can later be converted into a p-value by utilizing the t-distribution[18]. This negates our need to generate a population of random signals. With the ability to convert the correlation coefficient into a p-value, we can then easily set a cutoff which is directly comparable to other techniques for filtering genes such as the EDGE which has the option of reporting the p-value along with a q-value.

(1) $$\begin{array}{l} r=\frac{\left[N*\sum_{i=1}^{N}S_{1}(i)S_{2}(i)-\sum_{i=1}^{N}S_{1}(i)*\sum_{i=1}^{N}S_{2}(i)\right]}{N*\sqrt{\left[\sum_{i=1}^{N}S_{1}(i)S_{1}(i)-\sum_{i=1}^{N}S_{1}(i)*\sum_{i=1}^{N}S_{1}(i)\right]\left[\sum_{i=1}^{N}S_{2}(i)S_{2}(i)-\sum_{i=1}^{N}S_{2}(i)*\sum_{i=1}^{N}S_{2}(i)\right]}}\\ S_{1}\text{ = Values associated with Sample 1}\\ S_{2}=\text{Values associated with Sample 2}\\ N=\text{Total number of values in each Sample}\\ \text{r = Correlation Coefficient} \end{array}$$

While it is possible to rank all of the genes in a given array by this correlation score, we can also calculate a p-value associated with this correlation. Since the hypothesis underlying this method is that the inherent variation is visible given the limited number of replicates and inter-sample variability, the null hypothesis is that there is no inherent variation in the data, and all the variability is due to noise. Thus, a p-value will be established by generating a synthetic population of signals with the same number of replicates per time point as the microarray dataset. The synthetic signals that form the basis of comparison will have the same dimensions as the original dataset with the same number of replicates and time points, but the labels will be randomly permuted. The same LOOCV cross validation operation will be run on the synthetic data and the r-values computed for the synthetic signals. In a given dataset had 6 time points with 4 replicates each and the desired p-value was P < .05, 20 synthetic signals would be generated each with 6 time points and 4 replicates, and the LOOCV operation performed on this synthetic set. Within this population of genes, the highest level of correlation showed by a randomly generated signal was .55. Thus, the selection of high quality genes with P < .05 would entail the selection of genes which showed minimum correlation between its sub-sampled signals greater than .55.

### Data

While the proposed algorithm is applicable to biological signals in general, the data being utilized will be obtained from temporal gene expression experiments obtained via microarrays. These experiments are advantageous because they present a wide range of different signal to noise ratios, different number of replicates, as well as the ease in which it is possible to evaluate the biological relevance of the results in a quantitative fashion.

The data to be used are all publically available via the GEO database[19]. The data is publicly available from the GEO (Gene Expression Omnibus). The first dataset is an acute administration of corticosteroids upon a rat utilizing the RG-U34A microarray(GDS253)[1], the second dataset is a chronic administration of corticosteroids utilizing upon the RAE230A microarray(GDS972)[10], and the final dataset is a rat -burn model using the RG-U34A microarray(GDS599)[5]. These datasets were selected to illustrate the effect of different microarray platforms, different number of time points, as well as a different number of replicates per time point. The acute administration of corticosteroids along with the burn dataset were run off an older microarray platform the RG-U34A microarray, whereas the chronic administration of corticosteroids were run off the RAE230A array. In terms of signal length, the burn dataset consists of 5 time points, the acute corticosteroid dataset consisting of 17 time points, and the chronic administration of corticosteroids consisting of 11 time points. Finally, in terms of replicates, the burn dataset consists of 2 replicates per time point, the acute corticosteroid administration consists of anywhere from 2–4 replicates per time point, and the chronic administration of corticosteroids consists of 4 replicates per time point.

Based upon our two initial guiding principles that a lower coefficient of variance should lead to more accurately measured signals as well as the fact that increasing the number of replicates should lead to more accurately measured signals, we hypothesize that the burn dataset (GDS599) should show the greatest difference in the clustering performance due to its few replicates as well as due to its older experimental platform while the dataset consisting of the chronic administration of corticosteroids should show the least amount of difference because of improvements in the microarray itself as well as due to the greater number of replicates.

### Assessing the impact of high quality signals

While the motivation behind utilizing this filtering technique was to improve the confidence one has in the mathematical models derived from temporal biological data, such confidence is difficult to quantify without conducting additional experiments to validate a generated mathematical model. However, because the data used in the evaluation consists of high throughput gene expression profiles, a surrogate metric can be used. For temporal gene expression profiles, one of the primary hypotheses is that groups of genes with similar temporal progressions of their gene expression profiles will have similar functionalities. This assessment is to quantify the effect high quality signals have upon clustering. While there are numerous methods to assess the quality of a given clustering result such as external clustering similarity[20], we have elected to utilize gene ontology enrichment to expand the scope of comparison away from only assessing the clustering of mean expression values to a measure that is more grounded in the biological conclusions which a researcher would draw from the data.

In our case, we have elected to use the clustering package cluto[20], with the default parameters as a representative clustering approach. Therefore, with an increase in quality of clustering due to better signals, it should be possible to see an associated improvement in the enrichment[21,22]. Gene Ontology enrichment is conducted by utilizing the hypergeometric distribution as given in **(2)**. The ontologies themselves are obtained from the Affymetrix Annotations provided with each individual microarray. This hypergeometric distribution essentially calculates the probability that a subset of genes has been selected from an overall population. To evaluate the overall quality of a given enrichment, the metric will be the percentage of identified ontologies which have been selected as enriched. It is hypothesized that if the clustering is more reliable, then there should be a lower number of ontologies which had been spuriously included due to ambiguities within the signals.

(2) $$\begin{array}{l} P=1-\sum_{k=1}^{n}\frac{\left(\begin{array}{c} k\\ i \end{array}\right)\left(\begin{array}{c} m-k\\ N-i \end{array}\right)}{\left(\begin{array}{c} m\\ N \end{array}\right)};\\ \text{n = number of times the ontology appears in a given cluster}\\ \text{i = number of genes in a given cluster}\\ \text{N = total number of genes in the dataset}\\ \text{m = number of times the ontology appears in the dataset} \end{array}$$

Given that the initial claim of the manuscript is that it is important to select for genes which show not only significant differential expression, but also genes which show accurately measured expression profiles, thus we have elected to compare the performance of the proposed LOOCV algorithm vs. a standard method for selecting genes based upon differential expression EDGE[7].

One of the difficulties with this assessment is that the evaluation of gene enrichment is dependent upon the number of clusters with the data is partitioned into. Determining the number of clusters itself is an open area of research, and thus it is difficult to determine the proper number of clusters present within the data. Therefore, instead of focusing upon the number of clusters present in the data, the evaluation will be conducted over a continuum of different cluster numbers. It is hypothesized that if the filtering has been successful, then the percentage of significantly enriched ontologies will be greater for any given cluster number.

## Authors' contributions

IPA designed the study; EY performed the analysis and conducted all calculations. All authors read and approved the final manuscript.
